# Cross‐Species Insights into Cognition: Translating Findings from Mice to Humans at‐risk for Alzheimer's Disease

**DOI:** 10.1002/alz70855_099822

**Published:** 2025-12-23

**Authors:** Taylor W. Schmitz

**Affiliations:** ^1^ Schulich Medicine and Dentistry, London, ON, Canada; Robarts Research Institute, London, ON, Canada

## Abstract

**Background:**

The cross‐species translation of cognitive testing is a critical approach for advancing our understanding of Alzheimer's disease (AD). Mouse models of AD enable causal mechanistic insights into how genetic and biological factors influence cognitive decline. Recent developments in touchscreen‐based cognitive tasks allow for consistent testing paradigms in both humans and mice, bridging the gap between preclinical and clinical research.

**Method:**

Through an ongoing collaboration between Western and McGill Universities, we are leveraging homologous touchscreen tests of cognition in parallel studies of next‐generation mouse models of AD and humans at risk for AD. Mouse models with humanized knock‐in genes (amyloid, tau, ApoE ε3 or ε4) are being used at Western University to explore how genetic factors interact with age and sex to influence executive function. These experiments use the Continuous Performance Task (CPT), a sensitive touchscreen‐based probe of selective attention.

**Result:**

Since 2021, we have been integrating the CPT into the longitudinal PREVENT‐AD study led by McGill University and the Douglas Research Centre. Over 425 individuals (aged 60–80 years) with intact cognition at baseline and family history of AD are enrolled in PREVENT‐AD. Since 2011, this cohort has contributed a comprehensive dataset that includes multimodal neuroimaging, cerebrospinal fluid biomarkers, neurosensory measures, and standard cognitive assessments. We have acquired CPT in >220 individuals, with longitudinal data collected from approximately 80 of these participants and counting.

**Conclusion:**

This talk will explore the integration of touchscreen‐based cognitive testing into both human and mouse studies and its role in elucidating the mechanisms of AD risk. I will discuss how PREVENT‐AD human and mouse touchscreen CPT data can shed light on the interaction of ApoE genotype, age, and sex in early cognitive dysfunction. Additionally, I will explore whether touchscreen tests of cognition differ from standard neuropsychological tests in their sensitivity to early AD‐related decline. Finally, I will outline plans to expand the use of touchscreen tests within PREVENT‐AD and other large‐scale consortia. By linking genetic risk, cognitive performance, and neurobiological markers, this cross‐species approach will advance our understanding of AD's earliest manifestations and support the development of targeted interventions.

